# Girls play basketball too? A study of the mechanisms of traditional social gender consciousness on female participation in contact leisure sports

**DOI:** 10.3389/fpsyg.2024.1454003

**Published:** 2024-10-09

**Authors:** Yushan He, Guibin Su, Lujuan Wang, Haonan Qian

**Affiliations:** ^1^School of Martial Arts, Henan University, Kaifeng, Henan, China; ^2^Harbin Sport University, HarBin, Heilongjiang, China; ^3^Mudanjiang Normal University, Mudanjiang, Heilongjiang, China; ^4^School of Marxism, Henan University, Kaifeng, Henan, China; ^5^Department of Physical Education, Hanyang University, Seoul, Republic of Korea

**Keywords:** Women’s sports, traditional social gender consciousness, contact leisure sports, barriers to participation in leisure sports, gender bias, gender stereotypes

## Abstract

**Background:**

With the development of women’s sports, the mechanism of barriers to women’s leisure sports participation has attracted extensive attention from the academic community. Despite its significant impact, there are few empirical studies on intra-personal barriers. Consequently, a structural equation model was established to examine the relationships between traditional social gender awareness, gender bias, gender stereotypes, and barriers to participation in leisure sports.

**Methods:**

In this study, a total of 508 questionnaires were collected and analyzed using AMOS 24.0 software for structural equation modeling. After model testing, the relationships between the variables were examined.

**Results:**

The results of the statistical analyses indicated that traditional gender awareness could serve as an antecedent variable for barriers to participation in leisure sports, and that gender stereotypes mediated the relationship between traditional gender awareness and barriers to participation in leisure sports. The study also concluded that gender bias could not mediate the relationship between traditional gender awareness and participation barriers in leisure sports, but gender bias and gender stereotypes could act as chain mediators in the process of the influence of traditional gender awareness on barriers to contact leisure sports.

**Discussion:**

This study emphasizes the need for women to break down the traditional social gender awareness, gender bias, gender stereotypes, and other intra-personal barriers when engaging in leisure sport participation. According to this study, the promotion of sustained and healthy development of women’s sport requires the breaking down of traditional social gender awareness education and the creation of more gender-inclusive sports policies and environments. The significance of the study is that by proposing and confirming the internal participation barriers to women’s participation in contact leisure sports, it will lead to the ideological liberation of women’s gender perspectives, so that they can break down the participation barriers to contact leisure sports, participate in sports activities, and enjoy the right to play sports on an equal footing.

## 1 Introduction

Since the 21st century, the disparity in sports participation between men and women has gradually decreased, and equality has increased (Grabmüllerová, 2022). However, doubts about women’s participation in contact leisure sports persist. Contact leisure sports involve physical contact and collisions between participants, chosen during leisure time (Mlv et al., 2023). When women participate in basketball, they often hear, “Girls play basketball too?” “How can a girl be good at basketball?” and other gender-biased comments, while women who participate in more contact sports such as Taekwondo and Shotokan face even more gender-biased comments. In a survey of primary and secondary school children, 45% reported witnessing gender discrimination in sports. One child said, “Girls cannot tackle in school; they have to play flag football because it’s gentler” (Brown et al., 2011). The discourse around “male-dominated sports” significantly impacts women’s willingness to participate in contact leisure sports, leading to severe gender inequality. This study aims to theoretically explain the mechanisms behind this social phenomenon by investigating women’s participation in contact leisure sports. Enhancing equity in sports participation and creating a more inclusive environment for women will promote social progress and improvement.

Current research into the reasons for women’s low participation in contact leisure sport covers the following areas: past experience, knowledge of the sport, and environmental influences, on the basis of which some studies have also suggested barriers from a broader perspective, including insufficient coaching expertise, lack of time and energy, lack of engaging sports activities, and negative sports experiences (Hull et al., 2021). Most of the barriers to sport participation that previous studies have focused on are objective factors, but few studies have considered and argued the causes of barriers to women’s participation in sport from the perspectives of traditional gender consciousness, gender bias, and gender stereotypes, of which traditional gender consciousness refers to the fact that people’s awareness and evaluation of different genders’ social status, rights and responsibilities, and gender relations tend to be in the traditional gender culture (Ridgeway, 2011), gender bias refers to unfair attitudes towards a particular gender and its individual members, with negative, pre-judgmental characteristics (Myers, 1994), and gender stereotyping refers to a fixed, oversimplified, and stereotypical notion of how males and females should act, behave, and what roles they should play in society (Eagly et al., 2012). Most of the above studies are qualitative research on factors influencing participation in contact sports. They have not analyzed the mechanisms between traditional gender consciousness and participation in contact leisure sports. There is a lack of empirical research on the relationships between variables affecting women’s participation in such sports. This study aims to provide insights and references for the positive development of women’s sports through exploration and empirical research.

This study takes into account the fact that traditional gender consciousness may influence the formation of barriers to women’s participation in contact leisure sports, and that gender bias and gender stereotypes may also play a role in the formation of barriers. Based on this, traditional gender consciousness will be selected as the independent variable, barriers to participation in leisure sports as the dependent variable, and gender bias and stereotypes as mediating variables. A structural equation model will be used to examine the path relationships among these four variables. The results of this study aim to provide references for research on the mechanisms influencing women’s sports participation. Additionally, it seeks to break gender bias in women’s sports participation and promote the equalization of ideas and concepts in sports participation.

## 2 Theoretical background and research model

### 2.1 Literature review

#### 2.1.1 Barriers to participation in leisure sports

Barriers to participation in leisure sports refer to factors that limit or prevent participation in leisure sports activities aimed at relaxation, entertainment, fitness, stress relief, and excitement (Castro et al., 2021). The antecedents of barriers to participation in leisure sports are divided into three progressively advancing parts: intra-personal barriers, inter-personal barriers, and structural barriers. Intra-personal barriers refer to limitations formed by individual psychological and cognitive factors; inter-personal barriers refer to factors related to others, such as family attitudes and lack of companions; structural barriers refer to external factors like transportation, time, and money (Crawford et al., 1991). Research on structural barriers is relatively abundant. For example, married women exhibit high levels of structural barriers in indoor sports (Kim et al., 2020), and company employees face time and space limitations leading to barriers to participation in leisure sports (Eberth and Smith, 2010). Both intra-personal and inter-personal barriers have been studied in relation to different groups (Jackson and Scott, 2005; Son et al., 2009). Additionally, studies have shown a significant negative correlation between barriers to participation in leisure sports and active sports participation (Zhou et al., 2021). Empirical research has long confirmed that women face more barriers to participation in leisure sports than men (Jackson and Henderson, 1995), but previous studies have not specifically investigated barriers to participation for women participating in contact sports.

#### 2.1.2 Traditional gender consciousness

Traditional gender consciousness refers to the perception and evaluation of social status, rights, responsibilities, and gender relations that lean towards traditional gender norms. In contrast, modern gender consciousness emphasizes gender equality and respect for individual feelings and rights (Ridgeway, 2011). Research on traditional gender consciousness focuses on social surveys and empirical studies. Some studies have shown that women’s traditional gender consciousness significantly affects their ability to acquire and control resources (Parveen, 2007). Quantitative research has also indicated that the level of traditional gender consciousness may be an important factor influencing the work environment and professional distribution (Andersson et al., 2012). Previous studies have demonstrated that traditional gender consciousness significantly influences the participation of different genders in activities, deeply affecting individual behavior choices as a key cognitive factor.

#### 2.1.3 Gender bias

The researcher defines gender bias as an unjust attitude towards a specific gender and its individual members, characterized by negativity and preconception (Myers, 1994). Gender bias is divided into benign gender bias and hostile gender bias. Benign gender bias refers to restricting women to traditional roles by praising them for being gentle and kind, while hostile gender bias refers to showing hostility, rejection, and devaluation towards non-traditional women who defy traditional gender roles (Glick and Fiske, 1999, 2001, 2018). Previous studies have confirmed that unreasonable cultural backgrounds and values (Hofstede, 2001), educational environments (Pomerantz and Ruble, 1998), information dissemination (Ward, 2003), political systems (Lee et al., 2016), and economic factors (Bank W, 2012) all contribute to the formation of gender bias. The formation of gender bias significantly affects women’s workplace and academic environments (Roper, 2019). It is also important to note that gender bias does not exist solely towards women; it also affects men (Helmer et al., 2017).

#### 2.1.4 Gender stereotypes

Gender stereotypes refer to fixed, overly simplified, and generalized beliefs about how men and women should behave, perform, and what roles they should play in society (Eagly et al., 2012). According to social role theory, gender stereotypes stem from the different social role distributions of men and women in the family and workplace (Koenig and Eagly, 2014). Gender stereotypes not only describe typical differences between men and women but also dictate what men and women should be like and how they should behave in different areas of life (Nicholson et al., 2013). Due to differences in the ways society educates the two genders, the emphasis on different social roles and power positions increases with age, further amplifying the impact of gender stereotypes (Eagly and Wood, 2013).

### 2.2 Theoretical framework

The ABC model of attitudes reveals the relationship between affect, behavior tendencies and cognition in consumers. This model posits that attitudes are composed of three components: cognition, affect, and behavior. “Cognition” refers to the beliefs held about an attitude object; “affect” refers to the feelings and emotions towards the attitude object; and “behavior” refers to the intention or actual behavior towards the attitude object. In this model, “cognition” forms the basis for “affect” and “behavior”; “affect” acts as the core of attitude, serving as a mediator between “cognition” and “behavior,” while “behavior” is the result of the interaction between “cognition” and “affect” (Rosenberg, 1960). Building on this model, Solomon and others explained the hierarchical effects, showing different relationships between affect, behavior and cognition in consumer decision-making under standard learning hierarchy, low-involvement hierarchy, and experiential hierarchy (Solomon et al., 2012).

### 2.3 Hypotheses deduction

The behavior of refusing to participate in contact leisure sports is caused by the individual’s evaluation of women participating in contact leisure sports, and this evaluation stems from the individual’s cognition of the event (Ellis and Dryden, 2007). From the perspective of gender roles, women who identify with the inherent gender differences between men and women and the traditional division of social roles for women will have a traditional gender consciousness, and according to the ABC Attitude Model, these women are more likely to have the “cognition” of defining contact leisure sports as “men’s sports,” thus contributing to the formation of the “behavior” of barriers to sports participation. According to the ABC Attitude Model, these women are more likely to develop the “cognition” that defines contact leisure sports as “male sports,” thus contributing to the formation of “behaviors” that create barriers to sports participation. In addition to this, when individuals internalize traditional gender consciousness, they are more likely to develop an “affect” disposition to prejudice (Harmon-Jones and Mills, 2019), which can lead to gender bias, so it can be deduced that traditional gender consciousness may have a contributing effect on gender bias in contact leisure sport participation (Morais et al., 2022), as well as traditional societal gender consciousness that recognizes women’s tenderness and caring qualities more than women’s competence and assertiveness, which contributes to the formation of gender stereotypes of women (Eagly et al., 1992; Heilman, 2001). As a result, the following are our theories:

*H1a*: Traditional social gender consciousness is positively correlated with barriers to participation in leisure sports.

*H1b*: Traditional social gender consciousness is positively correlated with gender bias.

*H1c*: Traditional social gender consciousness is positively correlated with gender stereotypes.

Studies show that doing sports might be hampered by gender prejudice, especially in youth. As a result, we speculate that gender prejudice in women’s sports may also contribute to the establishment of barriers in contact leisure sports (Chalabaev et al., 2013). We also note that individuals are more likely to be influenced by gender stereotypes while making decisions about social learning. Applying this to women’s sports, we can hypothesize that barriers on contact leisure sports may also come from the gender stereotypes held by female participants (Cunningham et al., 2023). Additionally, there are certain connections between gender bias and gender stereotypes. Gender bias focuses more on negative perceptions of a particular group, whereas gender stereotypes involve expectations about behaviors or characteristics based on gender roles, which can be either positive or negative. People tend to seek out, remember, and trust information that aligns with their existing beliefs or biases, while ignoring or questioning information that contradicts them (Oswald and Grosjean, 2004). Therefore, when people hold biases against a particular gender, they may be more likely to accept and reinforce information that aligns with these biases, thereby deepening their gender stereotypes. Therefore, we propose the following hypotheses:

*H2a*: Gender bias is positively correlated with barriers to participation in leisure sports.

*H2b*: Gender stereotypes is positively correlated with barriers to participation in leisure sports.

*H3*: Gender bias is positively correlated with gender stereotypes.

Exploring how traditional social gender consciousness influences the occurrence mechanism of women’s barriers to participation in leisure sports, gender bias may serve as a mediating variable connecting the two. In the ABC attitude model, women, due to their own “cognitive” awareness of traditional social gender consciousness, develop negative “affective” gender biases, and ultimately avoid choosing “masculine” contact leisure sports out of fear of violating traditional gender norms. This phenomenon can easily lead to a decline in women’s self-efficacy, thereby resulting in the deprivation of opportunities and hindrances to the development of women’s rights to participate in sports (Messner, 2002). Besides gender bias possibly acting as a mediating variable between traditional social gender consciousness and the barriers on contact leisure sports, gender stereotypes may also serve as a mediating variable between the two. Gender differences arise from the internalization of gender identity defined by cultural expectations and ideals. For instance, boys learn from a young age that expressing emotions through crying is not a characteristic they should exhibit (Spence et al., 1975). Such socialization patterns lead men and women to form gender stereotypes after being shaped by their environment, resulting in differences in their preferences. Previous research indicates that gender temperament identification is related to sports participation, and the temperament identification of female participants is mostly hermaphroditic or male (Clément-Guillotin and Fontayne, 2011). Female sports participants, at the standard learning level, when making behavioral decisions about whether to engage in contact leisure sports, go through a decision-making process from the cognition of traditional social gender consciousness to the affective bias of gender stereotypes, ultimately leading to the behavioral choice of refusing to participate in contact leisure sports, forming a “cognition-affection-behavior” relationship chain (Rosenberg, 1960).

Further research into the distinctions and connections between gender bias and gender stereotypes reveals that gender bias is an unstable emotional attitude resulting from cognitive biases about gender roles and relationships, whereas gender stereotypes are more stable emotional attitudes. When people hold biases against a particular gender, they may selectively view and remember information that aligns with these biases to maintain cognitive consistency, which also leads to the reinforcement of gender stereotypes (Cheng et al., 2022). In summary, women with traditional social gender consciousness are more likely to form gender biases, which, when transformed into more stable gender stereotypes, can positively influence the formation of barriers to participation in leisure sports. Therefore, we propose the following hypotheses:

*H4*: Gender bias mediates the effect of traditional social gender consciousness on barriers to participation in leisure sports.

*H5*: Gender stereotypes mediate the effect of traditional social gender consciousness on barriers to participation in leisure sports.

*H6*: Gender bias and gender stereotypes act as a chain mediation in the effect of traditional social gender consciousness on barriers to participation in leisure sports.

**Figure 1:**
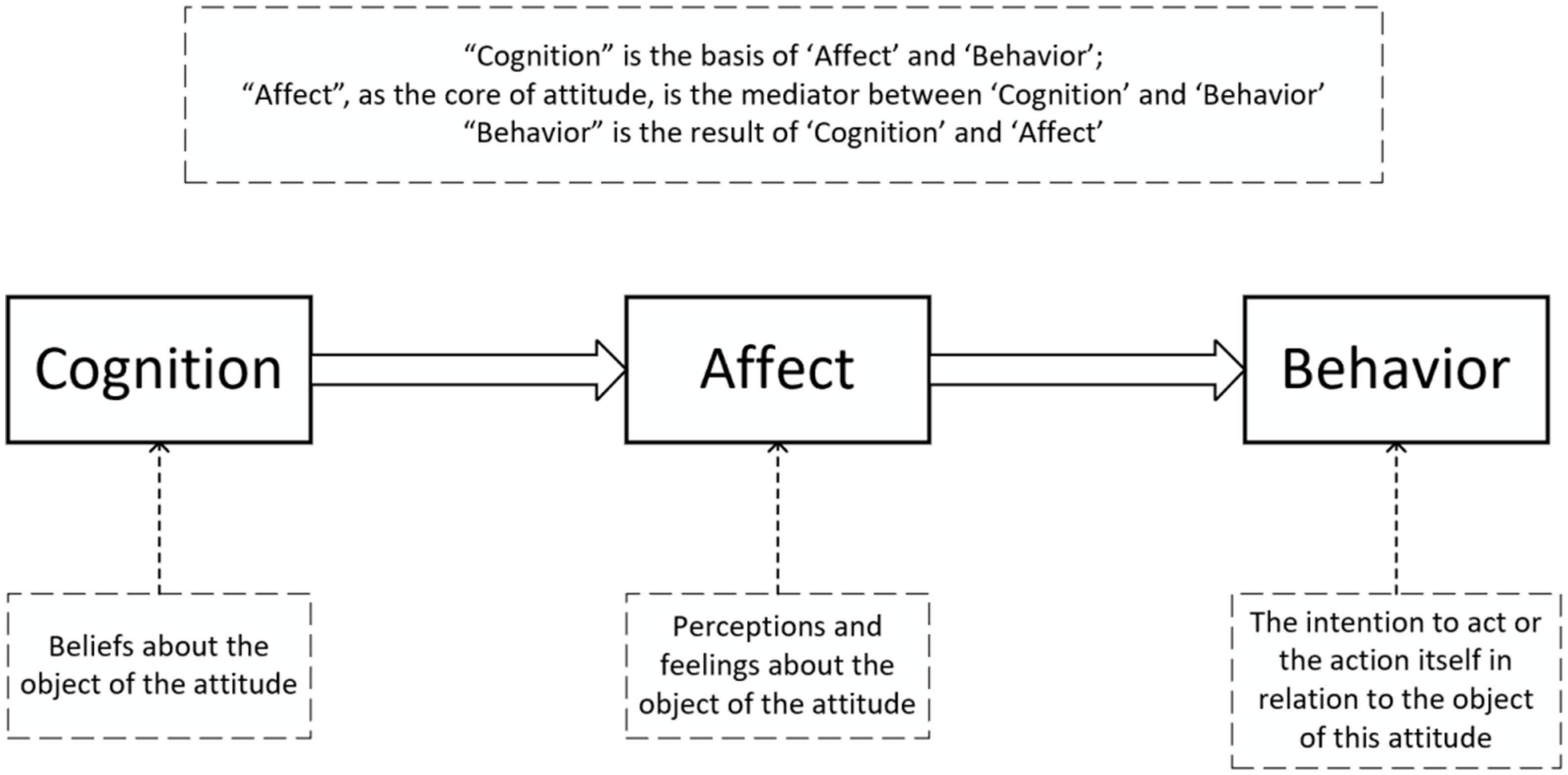
The ABC model of attitudes.

**Figure 2:**
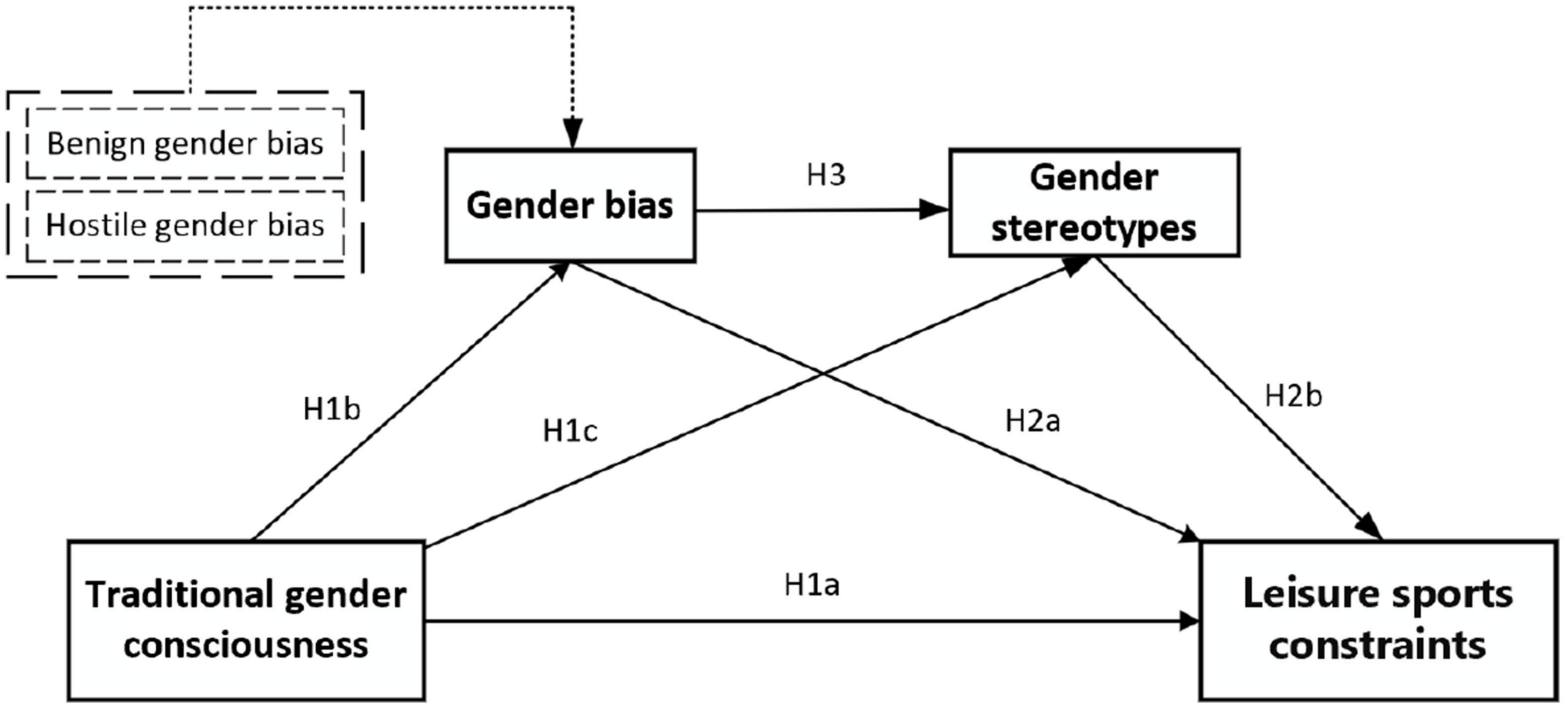
Conceptual mediation model and hypotheses.

Consistent with the literature review and hypothesis deduction in the previous sections, a diagram illustrating the model of this study is drawn, as shown in Figures 1, 2.

## 3 Method

### 3.1 Measurement of variables

The survey participants are women of various age groups. The questionnaire consists of three parts: the introduction, demographics, and the main body. The introduction explains the purpose of the research, includes an informed consent form, and instructs participants to answer based on their actual situations and thoughts. The study assures that all responses will be kept confidential and used solely for research purposes. The demographic section includes measurements of gender, age, occupation, and family monthly income. The main body is divided into two parts. The first part measures three variables: gender stereotypes, traditional social gender consciousness, and gender bias. The second part specifically measures the barriers on engaging in leisure sports.

The main part of the questionnaire uses a 5-point Likert scale (“1 = Strongly Disagree,” “2 = Disagree,” “3 = Neutral,” “4 = Agree,” “5 = Strongly Agree”). Some indicators are positively coded, assigning values of 1, 2, 3, 4, and 5 to “Strongly Disagree,” “Disagree,” “Neutral,” “Agree,” and “Strongly Agree,” respectively, while other indicators are reverse coded. Each variable in the questionnaire is evaluated by scholars participating in this research to assess the comprehensibility of the items. They suggest modifications and improvements to make the items clearer and more effective in measuring the intended variables. Subsequently, an expert panel reviews the items for any missing or redundant parts, conducts a semantic analysis of existing foreign questionnaires, and reduces the impact of different contexts on the content of the questionnaire items to ensure the results are more accurate and relevant (Table 1).

**Table 1:** Variables and measurement items.

Variable names	Scale measurement items	References
Gender stereotypes	GS1 Women’s and men’s sports performance are equally good (−)	Fennema and Sherman (1976) and Shujuan (2015)
	GS2 I find it hard to believe that a woman could be a sports genius	
	GS3 I believe women are as suitable as men to study sports (−)	
	GS4 Women usually need men’s help when encountering difficulties in studying sports	
	GS5 I believe women can solve problems in sports study just as men can (−)	
	GS6 I have more confidence in men’s sports performance compared to women’s	
	GS7 Women can perform as well as men in sports study (−)	
	GS8 Women who like sports make me feel a bit strange	
	GS9 Women also have good enough physical fitness to excel in sports (−)	
	GS10 Men are not inherently better than women in the field of sports (−)	
	GS11Combative sports belong to men, and sports that emphasize gracefulness belong to women	
Traditional gender consciousness	TGC1Men should prioritize their careers, while women should prioritize their families	Li and Fengping (2021)
	TGC2 Men are naturally more capable than women	
	TGC3 A good marriage is better than a good job	
	TGC4 During economic downturns, women employees should be laid off first	
	TGC5 Husbands and wives should equally share household chores (−)	
Gender bias	GB1Many women are actually seeking privileges under the guise of “equality,” hoping for favorable employment policies	Glick and Fiske (1996)
	GB2 Most women always think they are innocent or victims of gender discrimination	
	GB3 Most women are ungrateful for everything men do for them	
	GB4 Women try to control men to gain power	
	GB5 Women often exaggerate their problems at work	
	GB6 Once a woman gets a man’s commitment, she wants to keep him under control all the time	
	GB7 When women lose in fair competition with men, they always complain about gender discrimination	
	GB8 No matter how successful a man is, he is incomplete without a woman’s love	
	GB9 Women should be cherished and protected by men	
	GB10 Every man should have a woman he admires	
	GB11 A good woman should be cherished by her man	
	GB12 Women often have a higher sense of morality than men	
	GB13 Men should be willing to sacrifice themselves to provide financial security for women	
	GB14 Women often have higher cultural awareness and better taste than men	
Barriers to participation in leisure sports	LSC1 I would not participate in contact sports because I am too shy	Crawford and Godbey (1987)
	LSC2 If my friends do not support me in participating in contact sports, I am less likely to participate	
	LSC3 I am unlikely to participate in contact sports because it makes me feel physically uncomfortable	
	LSC4 The people I know usually cannot participate in contact sports with me because they lack the necessary funds	
	LSC5 The people I know usually cannot participate in contact sports with me because they have too many family obligations	
	LSC6 The people I know usually cannot participate in contact sports with me because they do not know which sports they can join with me	
	LSC7 The people I know usually cannot participate in contact sports with me because they lack the necessary sports skills	
	LSC8 If I have committed to other matters, I am unlikely to participate in contact sports	
	LSC9 If I cannot find suitable contact sports to participate in, I am unlikely to participate	
	LSC10 If the facilities or venues required for contact sports are inconvenient, I am unlikely to participate	
	LSC11 If I do not have time, I am unlikely to participate in contact sports	

(−) Indicates reverse items.

### 3.2 Data collection

After designing the questionnaire, 100 questionnaires were randomly distributed for a pretest and validation. Based on the feedback and pre-survey results, the questionnaire was adjusted and improved to finalize it.

From January 1 to February 20, 2024, a convenience sampling method was used, using the “Questionnaire Star” platform,^1^ which is the most authoritative and widely used online survey platform in China, and has the characteristics of distributing and collecting large sample sizes, and can be used to conveniently distribute questionnaires to various WeChat friends and communities. The entire questionnaire is in Chinese for easy understanding and selection by participants. After collecting the questionnaires, a total of 508 were distributed. Invalid questionnaires, including those with short completion times, more than 70% of identical responses, and male participants, were excluded. Finally, 503 valid questionnaires from 21 provinces in China were obtained, with an effective rate of 99%. Since this study’s subjects are limited to women, the questionnaire automatically ended if a participant selected male. The occupational distribution of female participants was: company employees (23.46%), students (18.09%), freelancers (6.96%), retirees (6.56%), government officials (6.36%), self-employed (4.97%), corporate managers (4.77%), medical personnel (4.37%), service industry workers (3.98%), laborers (3.78%), researchers (3.78%), agricultural workers (3.38%), legal professionals (3.18%), teachers (2.19%), full-time homemakers (0.2%), and other occupations (3.98%) (Table 2).

**Table 2:** Sample characterization statistics (*N* = 503).

Items	Amount	Frequency	Items	Amount	Frequency
Age			Current occupation		
≤18 years old	22	4.37%	Company employees	118	23.46%
18 ~ 25 years old	143	28.43%	Students	91	18.09%
26 ~ 30 years old	139	27.63%	Freelancers	35	6.96%
31 ~ 40 years old	54	10.74%	Retirees	33	6.56%
41 ~ 50 years old	58	11.53%	Government officials	32	6.36%
51 ~ 60 years old	61	12.13%	Self-employed	25	4.97%
Above 61 years old	26	5.17%	Corporate managers	24	4.77%
Gender			Medical personnel	22	4.37%
Female	503	100%	Service industry Workers	20	3.98%
Monthly household income (RMB)			Laborers	19	3.78%
≤5,000	86	17.1%	Researchers	19	3.78%
5,001 ~ 7,000	89	17.69%	Agricultural workers	8	3.38%
7,001 ~ 10,000	73	14.51%	Legal professionals	17	3.18%
10,001 ~ 13,000	137	27.24%	Teachers	16	2.19%
13,001 ~ 15,000	84	16.7%	Full-time homemakers	1	0.2%
≥15,001	34	6.76%	Other occupations	20	3.98%

### 3.3 Data results and analysis

#### 3.3.1 Reliability and validity testing

Reliability analysis is used in research to identify the reliability of quantitative data. Internal consistency coefficient (Cronbach’s *α*) is used to assess the consistency of each item in the questionnaire for the research variables. Generally, a value greater than 0.8 indicates ideal data reliability (Chin, 1998). In this study, the α value for the gender bias scale is 0.929, for the traditional social gender consciousness scale is 0.898, for the gender stereotype scale is 0.839, and for the barriers to participation in leisure sports scale is 0.897. All four scales have α values greater than 0.8, indicating high data reliability. In addition, the composite reliability (CR) values for each scale range from 0.839 to 0.93, indicating ideal reliability and demonstrating good reliability of the collected data.

Validity analysis mainly measures the structural validity of the scales, including convergent validity and discriminant validity. Overall structural validity first refers to the KMO value and Bartlett’s test of sphericity. The KMO value for the total scale is 0.974, and the *p*-value for Bartlett’s test of sphericity is 0.000, which is less than 0.05, indicating that the data collected by the scale has basic structural validity and can be subjected to factor analysis. On this basis, confirmatory factor analysis was used to measure the loading values of each item on the factors. The criteria for factor loading greater than or equal to 0.7 and average variance extracted (AVE) greater than 0.5 were used for testing. After optimizing the original scales, the following results were obtained: the gender bias dimension was optimized through two rounds, retaining items GB1 to GB7; the traditional social gender consciousness dimension was optimized through one round, retaining items GC1 to GC4; the gender stereotype dimension was optimized through one round, retaining items GS3, GS5, GS7, and GS9. The factor loadings of the optimized scale items ranged from 0.706 to 0.847, and the AVE values were all greater than 0.5, indicating good convergent validity of the research scales (Anderson and Gerbing, 1988). Discriminant validity was tested mainly by comparing the square root of the AVE value with the correlation coefficients between variables. If the square root of the AVE value is greater than any correlation coefficient between a pair of variables, it is considered to have discriminant validity (Fornell and Larcker, 1981). The measurement results in Table 3 indicate that all correlation coefficients between variables are less than the square root of the AVE, demonstrating good discriminant validity of the scales (Table 4).

**Table 3:** Confirmatory factor analysis and reliability and validity indicators.

Constructs	Factors	Mean	Factor loading	Reliability coefficient	CR	AVE	α value
Gender bias (GB)	GB1	3.28	0.794	0.921	0.930	0.655	0.929
	GB2	3.39	0.759	0.923			
	GB3	3.27	0.812	0.919			
	GB4	3.30	0.836	0.916			
	GB5	3.27	0.833	0.916			
	GB6	3.29	0.842	0.915			
	GB7	3.30	0.784	0.921			
Traditional gender consciousness (GC)	TGC1	3.22	0.847	0.86	0.898	0.688	0.898
	TGC2	3.25	0.810	0.876			
	TGC3	3.21	0.815	0.87			
	TGC4	3.06	0.845	0.866			
Gender stereotypes (GS)	GS3	3.94	0.721	0.808	0.839	0.567	0.839
	GS5	3.86	0.759	0.794			
	GS7	3.96	0.762	0.793			
	GS9	3.94	0.768	0.79			
Leisure sports constraints (PR)	LSC1	3.30	0.776	0.881	0.897	0.555	0.897
	LSC2	3.35	0.769	0.881			
	LSC3	3.50	0.732	0.884			
	LSC4	3.36	0.771	0.879			
	LSC5	3.51	0.738	0.883			
	LSC6	3.60	0.721	0.883			
	LSC7	3.58	0.706	0.885			

**Table 4:** Correlation coefficient and the square root of the average variance extraction.

	1	2	3	4
1 Traditional gender consciousness	**0.829**			
2 Gender bias	0.956***	**0.809**		
3 Gender stereotypes	0.112	0.199**	**0.753**	
4 Leisure sports constraints	0.875***	0.877***	0.371***	**0.745**

The bold values on the diagonal of the matrix are the square root of the Average Variance Extracted (AVE); the values below the diagonal are the correlation coefficients between the variables; *** indicates *p* < 0.001, ** indicates *p* < 0.01, * indicates *p* < 0.05.

#### 3.3.2 Structural model testing

To test the structural dimensions of traditional social gender consciousness, gender bias, social stereotypes, and barriers to participation in leisure sports, further confirmatory factor analysis was conducted on the scales of each dimension using AMOS 24.0 software, based on the results of the confirmatory factor analysis from the previous section.

**Figure 3:**
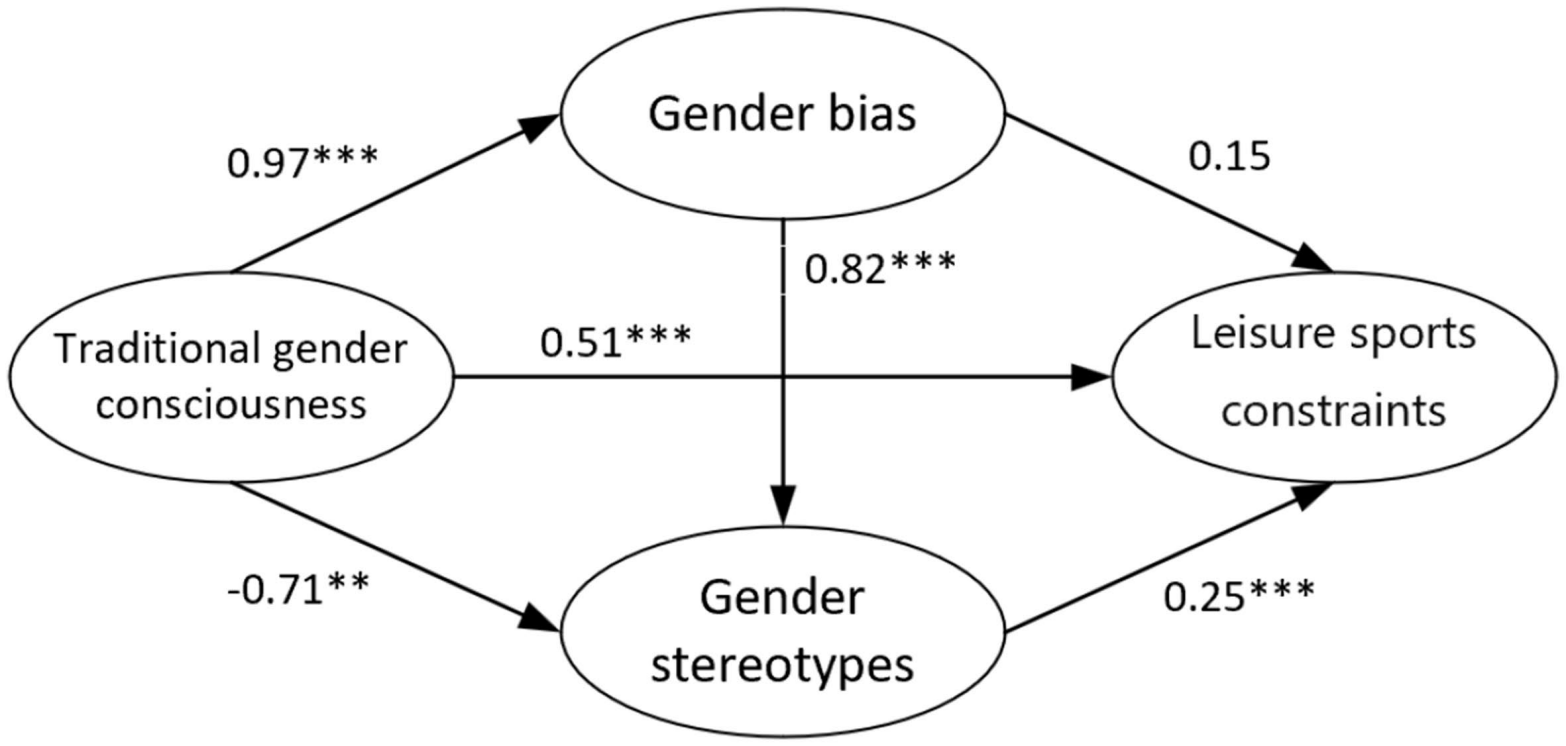
Structural model verification results.

In the validation model, the four variables of traditional social gender consciousness, gender bias, social stereotypes, and barriers to participation in leisure sports had the following indices: *X*^2^/df = 1.60 (<3), RMSEA = 0.035 (<0.05), and GFI, AGFI, NFI, IFI, and CFI values of 0.941, 0.926, 0.985, 0.984, and 0.984, respectively, all of which are greater than 0.9. This indicates a good overall fit between the validation model and the sample data (Hu and Bentler, 1999). Further analysis of the data reveals that the standardized path coefficients of traditional social gender consciousness to gender bias, gender stereotypes, and barriers to participation in leisure sports are 0.956, −0.907, and 0.657, respectively (Table 5). These coefficients are significant at the 0.001 and 0.01 levels. Since the measurement items for gender stereotypes are all reverse-coded, this demonstrates that traditional social gender consciousness has a direct positive impact on gender bias, gender stereotypes, and barriers to participation in leisure sports. The standardized path coefficient from gender bias to gender stereotypes is 1.066 (Table 5), significant at the 0.001 level, indicating that gender bias has a direct positive impact on gender stereotypes. There is no significant positive impact of gender bias on barriers to participation in leisure sports. The standardized path coefficient from gender stereotypes to barriers to participation in leisure sports is 0.258 (Table 5), significant at the 0.001 level, indicating that gender stereotypes also have a direct positive impact on barriers to participation in leisure sports (Figure 3).

**Table 5:** Structural model evaluation index and hypothesis test results.

Relationship hypothesis	Standardized path coefficients	*t* value	Test results
TGC → GB	0.956***	20.722	Support
TGC → GS	−0.907**	−2.811	Support
GB → GS	1.066***	3.22	Support
GB → LSC	0.198n.s.	1.129	Not supported
TGC → LSC	0.657***	3.732	Support
GS → LSC	0.258***	7.128	Support

*** Indicates *p* < 0.001, ** indicates *p* < 0.05, same below; n.s. indicates not significant.

#### 3.3.3 Mediation effect testing

To test the mediating effects of gender bias and gender stereotypes in the relationship between traditional social gender consciousness and barriers to participation in leisure sports, the method suggested by scholars was used (Jose, 2013). Bootstrap analysis was conducted with AMOS 24.0 software, setting the bootstrap sample size to 5,000 and the confidence level to 95%. Following the steps for testing specific effects, the total effects, direct effects, and indirect effects coefficients between the variables were obtained, along with their corresponding confidence intervals and significance *p*-values (Macho and Ledermann, 2011). Combining Tables 5, 6, traditional social gender consciousness has a direct impact on barriers to participation in leisure sports (0.673). Traditional social gender consciousness indirectly affects barriers to participation in leisure sports through gender stereotypes (−0.18). Given the significant direct impact of traditional social gender consciousness on barriers to participation in leisure sports, it can be concluded that gender stereotypes partially mediate the relationship between traditional social gender consciousness and barriers to participation in leisure sports. Additionally, traditional social gender consciousness can indirectly influence barriers to participation in leisure sports through the two variables of gender bias and gender stereotypes (0.202), forming a chain mediating effect. Therefore, hypotheses H1a, H2a, H1b, H1c, H3, H5, and H6 are confirmed, while H2b and H4 are not supported.

**Table 6:** Mediation effect test: bootstrap analysis.

Relationship paths	Total effects	Indirect effect	Confidence interval (95%)	*p*-value
TGC → LSC	0.673	–	0.591 ~ 0.758	0.000
TGC → GB → GS → LSC	–	0.202	0.069 ~ 0.537	0.0001
TGC → GB → LSC	–	0.145	−0.284 ~ 0.455	0.371
TGC → GS → LSC	–	−0.18	−0.506 ~ −0.047	0.005
GB → GS → LSC	–	0.208	0.071 ~ 0.543	0.001

## 4 General discussion

### 4.1 Theoretical contribution

The results from this study’s investigation of women’s participation in contact sports while rejecting female norms and being prone to teasing and rejection are consistent with previous research (Slater and Tiggemann, 2010). It is due to this social environment that these smart girls, although academically successful, usually only participate in one sport in a gender-specific manner (Schmalz and Kerstetter, 2006). This study explores the reasons behind such social phenomena, firstly, it proposes the role of traditional gender consciousness as an obstacle to women’s participation in contact leisure sports, and then it digs deeper into the content of traditional gender cognition that brings about a sense of alienation from sports, which has been summarized by previous studies (Ennis, 2000; Griffin, 1984), and proves that traditional gender consciousness of women will form an obstacle when they participate in “masculine” contact leisure sports. The empirical evidence that women’s traditional social gender consciousness creates barriers to participation in “masculine” contact leisure sports contributes to a deeper understanding of the impact of social ideology on women’s sport participation and provides a scientific basis for changing traditional gender perceptions. Secondly, based on the evidence that traditional gender consciousness positively influences gender bias and stereotypes (Eagly et al., 1992; Heilman, 2001), the study expands the scope of the research and the field of application to include women’s participation in contact leisure sports.

Additionally, this study also confirmed that when women internalize traditional social gender awareness, they are prone to forming gender bias and gender stereotypes under the influence of cognitive consistency. The process from “cognition” to “emotion” has been empirically tested (Harmon-Jones and Mills, 2019). Meanwhile, the study examined that gender bias does not have a direct impact on the formation of barriers to participation in leisure sports for female participants, whereas gender stereotypes have a positive impact on the formation of such constraints. This is somewhat similar to previous research which indicated that adolescents’ social learning choices are more influenced by gender stereotypes than by their own gender bias (Cunningham et al., 2023). The study also confirmed that the formation of gender bias deepens individuals’ gender stereotypes, further clarifying the logical relationships among the variables (Oswald and Grosjean, 2004).

Ultimately, traditional social gender awareness affects individuals’ perception of gender stereotypes, which in turn influences their perception of sports constraints (Nicholson et al., 2013). Gender stereotypes induced by traditional social gender awareness play a crucial mediating role in the sports domain, exacerbating individuals’ perception of barriers to participation in leisure sports. Interestingly, gender bias does not directly influence the perception of barriers to participation in leisure sports in this process, but rather indirectly affects it through gender stereotypes. This chain mediation effect reveals the subtle and complex relationship between gender bias and gender stereotypes, and how this relationship affects individuals’ perception and behavior in the sports environment (Cheng et al., 2022). The findings of this study are significant in explaining the phenomenon of gender differences in sports participation. Traditional social gender awareness, as a product of cultural traditions and socialization processes, has a profound impact on individuals’ participation in sports activities. By revealing the mediating role of gender stereotypes under the influence of traditional gender awareness, this study enriches the understanding of gender differences in sports participation and provides a new perspective for addressing gender inequality issues.

Compared to existing literature, this study found that the impact path of gender bias on sports barriers differs from previous research. Traditional views suggest that gender bias directly influences individuals’ perception of the sports environment (Smith and McCarthy, 2022). However, this study found that gender bias indirectly influences the perception of sports barriers by affecting gender stereotypes, which differs from traditional views. This indicates that the mechanism of gender bias may be more complex than previously thought, requiring further research to uncover its underlying mechanisms.

### 4.2 Practical implication

It can be seen that gender inequality has significant impacts in various fields. By conducting this study to clarify the formation mechanisms and principles of such phenomena, and then promoting gender equality awareness in the sports field through educational activities and media reports, traditional gender concepts in society can be changed, thereby reducing the barriers women face in participating in contact leisure sports. This study emphasizes the important role of gender stereotypes in influencing individuals’ perception of barriers to participation in leisure sports. Therefore, interventions targeting gender stereotypes become crucial. Educational and promotional activities can help break gender stereotypes, enhance individuals’ objective understanding of the sports environment, and thereby reduce the perception of barriers to participation in leisure sports. Since gender bias does not directly affect the perception of barriers to participation in leisure sports, merely reducing gender bias may not be sufficient to effectively increase women’s willingness to participate in sports. On the contrary, it is necessary to combine interventions on gender stereotypes to better improve women’s cognition and perception of the sports environment, thereby promoting their participation in sports.

Additionally, the research results indicate that the impact of traditional social gender awareness on individuals’ participation in sports activities is profound and needs to be addressed through institutional and policy reforms. Establishing sports policies and environments that embrace gender diversity, breaking the shackles of traditional gender roles, and providing more opportunities and resources for sports participation will help promote gender-equal sports development. At the same time, advocating for education that breaks traditional social gender awareness, making teachers more aware of the existence of social gender awareness, and reasonably guiding students’ traditional social gender awareness will help students make more confident and autonomous choices in accordance with their inner preferences in terms of subjects or careers. This study attempts to explain the mechanisms influencing the barriers to female participation in contact sports, verifying the existence of inter-relationships among the influencing factors, with the expectation of alerting stakeholders to and improving the current situation of female sports participation.

## 5 Limitations and future research

Although this study provides valuable contributions in revealing the causal relationship between traditional social gender awareness and barriers on women’s participation in contact leisure sports, there are still some limitations that offer directions for future research. Firstly, the sample of this study may have geographical and cultural limitations, as different geographical and cultural contexts can have varying impacts on traditional social ideologies and gender concepts. This study was conducted on women in the Chinese region. Future studies may consider expanding the sample to cover different regions and cultural backgrounds in order to obtain more comprehensive and diverse results. Future research can consider expanding the sample to cover different regions and cultural backgrounds to obtain more comprehensive and diverse results. Secondly, the variables included in the study’s predictive model are not comprehensive. Future research can consider adding other factors that may influence women’s participation in contact sports to more accurately analyze the complexity and interactions of the influencing factors.

Additionally, this study mainly focuses on validating the theoretical model and provides some suggestions for addressing cognitive and social phenomena. However, the effectiveness and feasibility of practical impacts still need further research and validation. Future research can employ experimental design or longitudinal studies to evaluate the effectiveness and practical benefits of promoting gender equality awareness and improving sports environment. Lastly, this study uses a cross-sectional design, which can only establish correlations and not causations. Future research can adopt longitudinal study designs to track changes in women’s participation in contact leisure sports and the evolution of influencing factors to better understand causality and dynamic processes.

In summary, future research can address the limitations of this study by expanding the sample, adding more comprehensive variables, evaluating the effectiveness of practical impacts, and adopting longitudinal study designs. These efforts will contribute to a deeper understanding of the barriers of traditional social gender awareness on women’s participation in contact sports and provide more reliable bases for formulating more targeted interventions.

## Data Availability

The original contributions presented in the study are included in the article/supplementary material, further inquiries can be directed to the corresponding author.

## Footnotes

^1^https://www.wjx.cn/
